# Fourier domain closed-form formulas for estimation of kinetic parameters in reversible multi-compartment models

**DOI:** 10.1186/1475-925X-11-70

**Published:** 2012-09-20

**Authors:** Gengsheng L Zeng, Dan J Kadrmas, Grant T Gullberg

**Affiliations:** 1 Utah Center for Advanced Imaging Research, Department of Radiology, University of Utah, 729 Arapeen Drive, Salt Lake City, Utah, 84108, USA; 2 Department of Radiotracer Development & Imaging Technology, Lawrence Berkeley National Laboratory, One Cyclotron Road, Mailstop: 55R0121, Berkeley, CA 94720, USA

**Keywords:** Kinetic parameter estimation, Dynamic imaging, Least-squares estimation, Nuclear medicine imaging, Compartment modeling, Fourier transform

## Abstract

**Background:**

Compared with static imaging, dynamic emission computed tomographic imaging with compartment modeling can quantify *in vivo* physiologic processes, providing useful information about molecular disease processes. Dynamic imaging involves estimation of kinetic rate parameters. For multi-compartment models, kinetic parameter estimation can be computationally demanding and problematic with local minima.

**Methods:**

This paper offers a new perspective to the compartment model fitting problem where Fourier linear system theory is applied to derive closed-form formulas for estimating kinetic parameters for the two-compartment model. The proposed Fourier domain estimation method provides a unique solution, and offers very different noise response as compared to traditional non-linear chi-squared minimization techniques.

**Results:**

The unique feature of the proposed Fourier domain method is that only low frequency components are used for kinetic parameter estimation, where the DC (i.e., the zero frequency) component in the data is treated as the most important information, and high frequency components that tend to be corrupted by statistical noise are discarded. Computer simulations show that the proposed method is robust without having to specify the initial condition. The resultant solution can be fine tuned using the traditional iterative method.

**Conclusions:**

The proposed Fourier-domain estimation method has closed-form formulas. The proposed Fourier-domain curve-fitting method does not require an initial condition, it minimizes a quadratic objective function and a closed-form solution can be obtained. The noise is easier to control, simply by discarding the high frequency components, and emphasizing the DC component.

## Introduction

Dynamic emission computed tomographic imaging can measure the kinetics of the tracer’s distribution and exchange with body tissues. Using quantitative analysis techniques such as compartment modeling [1-4], dynamic imaging can quantify *in vivo* physiologic and metabolic processes, providing more information regarding underlying molecular disease processes than can be obtained from static imaging. However, the fitting of compartment models to dynamic imaging data can be computationally demanding and can have problems with local minima.

A number of techniques for kinetic parameter estimation have been studied and are in use today, generally offering a tradeoff between computation time, robustness of fit, and flexibility with differing sets of assumptions. Perhaps the most robust—but also most computationally demanding—approach for estimating individual rate parameters for multi-compartment models is classic nonlinear least-squares estimation [5]. Here, a least-squares or weighted least-squares objective function is iteratively minimized to obtain best-fit kinetic parameter estimates. Numerous curve-fitting algorithms have been investigated for this application, including derivative-based or downhill simplex methods [5], ridge-regression [6-11], simulated annealing [12], and even discretized exhaustive search paradigms. The best results are obtained when accurate weighting is applied, though determination of the best weights is itself a challenging problem that depends on many variables including the reconstruction algorithm used [13]. Unfortunately, the least-squares objective function for multi-compartment models with noisy data is ill-formed, containing broad shallow valleys and (potentially) local minima. As such, careful implementation of the curve-fitting algorithm with extensive iteration and handling of local minima is required to confidently find the global minimum. This results in computational-demands that, while reasonable for fitting individual time-activity curves, may become impractical for voxelwise parametric imaging where millions of fits need to be performed.

The conventional least-squares curve-fitting method is to match the measured time-activity-curve with the calculated solution of the ordinary differential equations. The solution is in the form of a non-linear function of time and the kinetic parameters. It is neither in the ODE form or the integral form. Much of the difficulty in the curve-fitting approaches is due to the presence of nonlinear terms in the solution to the compartment modeling equations. Significant efforts have been made to “linearize” the problem, with milestones including the weighted integration method [14-16], linear least-squares (LLS) and generalized linear least-squares (GLLS) methods [17-20]. Such methods are based on integrating the compartment modeling equations to obtain linear systems of equations relating the rate parameters (or combinations thereof) to integrals of the time-activity curves and blood input functions. Though the image noise is not correlated between timeframes, integrating the time-activity curves in time gives equation errors that are not statistically independent, and that can bias the results (*e.g.* LLS); this correlation-induced bias can be reduced or eliminated by the iterative auto-regressive filtering technique of GLLS. More recently, approaches employing temporal basis functions to reduce noise and improve parameter estimation have also been studied [21-26]. Performance comparisons of these methods have been performed by Feng *et al.*[27] and more recently by Dai *et al.*[28]. Overall, nonlinear least-squares was found to provide the most robust parameter estimates, though this approach is also the most computationally intensive and initial condition dependent. Of the fast approaches, GLLS performed well for lower noise data, but may exhibit large bias and poor precisions when the noise level is high. Certain basis function approaches appear promising, though they are currently slower than GLLS and less thoroughly investigated.

This work offers a new perspective to the compartment model fitting problem where Fourier linear system theory is applied to derive closed-form formulas for estimating kinetic parameters for the two-tissue compartment model. This paper is an extension of our conference presentation at 2011 IEEE Medical Imaging Conference [29]. It builds on our related work developing closed-form solutions in the time domain [30], where the continuous-time differential equation is transformed into a discrete-time difference equation by using the exact continuous-time expression of the compartment activity. This time-domain approach is fundamentally different from time-domain curve-fitting approaches, and thus will likely have very different characteristics in terms of accuracy, speed, noise propagation and precision.

This paper introduces Fourier-domain closed-form solutions for the estimation of kinetic parameters for the two-tissue compartment model with 4 rate parameters. The similarity of the time-domain method and this Fourier-domain method are in the closed-form strategy to find the optimal solution of an objective function. Implementation procedures of the approach are discussed, and initial computer simulations are performed demonstrating application of the technique. The unique feature of the proposed method is that the formulation is in the Fourier domain, where the high-frequency noisy components can be readily discarded, and exponential functions never appear.

## Methods and results

This section presents the standard method of non-linear fitting to estimate the kinetic parameters, points out its potential deficiencies of the dependency on the initial conditions, and develops a closed-form Fourier domain method that is independent of the initial conditions. The result of the proposed Fourier domain estimation method is close to the true solution and can be used as the initial condition for the standard iterative method for refining.

### The standard iterative curve-fitting method

**Figure 1:**
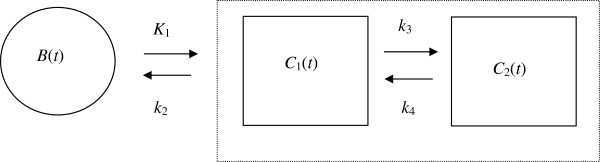
A general two-compartment-model.

Figure 1 shows a generic two-tissue compartment model with four rate constants: *K*_1_, *k*_2_, *k*_3_ and *k*_4_. The model can be described by two first-order differential equations:

(1) $$\frac{dC_{1}\left(t\right)}{dt}=\left(-k_{2}-k_{3}\right)C_{1}\left(t\right)+k_{4}C_{2}\left(t\right)+K_{1}B\left(t\right)\text{,}$$

(2) $$\frac{dC_{2}\left(t\right)}{dt}=k_{3}C_{1}\left(t\right)-k_{4}C_{2}\left(t\right)\text{.}$$

The above two equations are referred to as the state equations in system theory. The time-activity curve for the blood input is *B*(*t*). There are two tissue compartments *C*_1_(*t*) and *C*_2_(*t*); these two compartments are not individually accessible and their time-activity curves cannot be individually measured; however, the sum of them, *C*(*t*) defined as

(3) $$C\left(t\right)=C_{1}\left(t\right)+C_{2}\left(t\right)$$

can be measured. Equation (15) is called the output equation in system theory. Combining (1), (2) and (3) yields a second order differential equation:

(4) $$\frac{d^{2}C\left(t\right)}{dt^{2}}=x_{1}\frac{dB\left(t\right)}{dt}+x_{2}B\left(t\right)+x_{3}\frac{dC\left(t\right)}{dt}+x_{4}C\left(t\right)$$

with

(5) $$\left\{ \begin{array}{l} x_{1}=K_{1}\\ x_{2}=K_{1}\left(k_{3}+k_{4}\right)\\ x_{3}=-\left(k_{2}+k_{3}+k_{4}\right)\\ x_{4}=-k_{2}k_{4} \end{array}\text{.}\right.$$

The solution of (4) can be expressed as two convolution terms:

(6) $$C\left(t\right)=\alpha_{1}\int_{0}^{t}e^{s_{1}\left(t-\tau \right)}B\left(\tau \right)d\tau +\alpha_{2}\int_{0}^{t}e^{s_{2}\left(t-\tau \right)}B\left(\tau \right)d\tau \;\text{for}\;t\geq 0\text{,}$$

where

(7) $$s_{1}=\frac{x_{3}+\sqrt{x_{3}^{2}+4x_{4}}}{2}=\frac{-\left(k_{2}+k_{3}+k_{4}\right)+\sqrt{{\left(k_{2}+k_{3}+k_{4}\right)}^{2}-4k_{2}k_{4}}}{2}\text{,}$$

(8) $$s_{2}=\frac{x_{3}-\sqrt{x_{3}^{2}+4x_{4}}}{2}=\frac{-\left(k_{2}+k_{3}+k_{4}\right)-\sqrt{{\left(k_{2}+k_{3}+k_{4}\right)}^{2}-4k_{2}k_{4}}}{2}\text{,}$$

(9) $$\alpha_{1}=x_{1}\cdot \frac{s_{1}+x_{2}/x_{1}}{s_{1}-s_{2}}=K_{1}\cdot \frac{s_{1}+\left(k_{3}+k_{4}\right)}{s_{1}-s_{2}}\text{,}$$

(10) $$\alpha_{2}=x_{1}\cdot \frac{s_{2}+x_{2}/x_{1}}{s_{2}-s_{1}}=K_{1}\cdot \frac{s_{2}+\left(k_{3}+k_{4}\right)}{s_{2}-s_{1}}\text{,}$$

The standard iterative parameter estimation method is to minimize the objective function:

(11) $$F={\left\|C_{\mathrm{measured}}\left(t\right)-C_{\mathrm{model}}\left(t\right)\right\|}^{2}\text{,}$$

which measures the discrepancy between the measured tissue time-activity-curve *C*_*measured*_(*t*) and the estimated time-activity-curve *C*_*model*_(*t*) evaluated by (6).

More precisely, $F=\sum_{i}w_{i}{\left[C_{\mathrm{measured}}\left(t_{i}\right)-C_{\mathrm{mod}el}\left(t_{i}\right)\right]}^{2}$, where *C*_*measured*_(*t*_*i*_) is the measured activity count at time *t*_*i*_ and *w*_*i*_ is the associated reciprocal variance. Also note *C*_*model*_(*t*_*i*_) need not be in the form of a convolution integral. We can alternately use an ODE-solver in conjunction with curve-fitting to compute *C*_*model*_(*t*_*i*_) directly from the (1), (2), and (3) as needed in each curve-fitting iteration.

Since the gradient of the objective function *F* in (11) is non-linear with respect to the unknown parameters: *K*_1_, *k*_2_, *k*_3_ and *k*_4_, the result of the iterative algorithm will in general depend on the initial conditions. This phenomenon will be illustrated by two examples in the next section. After the examples, we will present a Fourier domain estimation method to solve the problem of dependency on the initial condition. The Fourier domain estimation method is the main contribution of this paper.

### Numerical examples of the standard iterative curve-fitting method

In the following computer simulations, the blood input functions were in the form of [31]:

(12) $$\hat{B}\left(t\right)=\left(A_{1}t-A_{2}-A_{3}\right)e^{-\lambda_{1}t}+A_{2}e^{-\lambda_{2}t}+A_{3}e^{-\lambda_{3}t},t\geq 0\text{,}$$

where *A*_1_ = 22.24, *A*_2_ = 8.36, *A*_3_ = 0.10, *λ*_1_ = 21.28 min^-1^, *λ*_2_ = 7.71 min^-1^, and *λ*_3_ = 0.37 min^-1^. The blood and tissue time activity curves were non-uniformly sampled as 30×5.sec., 20×10 sec., 10×30 sec., 10×60 sec., 10×150 sec., and 3×300 sec. At each sampling time, the activity was integrated over a 60-second time window. The total scanning time was about one hour (*i.e.*, 3650 seconds).

Measurements as “30×5 sec” means that 30 samples are taken and the samples are 5 sec apart between adjacent samples. Each sample is a continuous time integral of the time-activity-curve, and the integration time interval is 60 sec. The upper limit of the time interval is the sampling time instant. Therefore, the sampled signal is a collection of overlapped time integrals of the actual time-activity-curve. Even when the gap between adjacent samples is 300 sec, the integration time interval is still 60 sec, and in this case there is no overlap for time integral intervals for the samples.

The purpose of time integration is to reduce noise. Modern nuclear medicine scanners can acquire data in list-mode, in which data are acquired continuously with a non-discretized time stamp for each event. After the list-mode data are acquired, the discrete samples are formed for image reconstruction. When the discrete samples are formed, the time integral (i.e., count summation) is performed.

**Figure 2:**
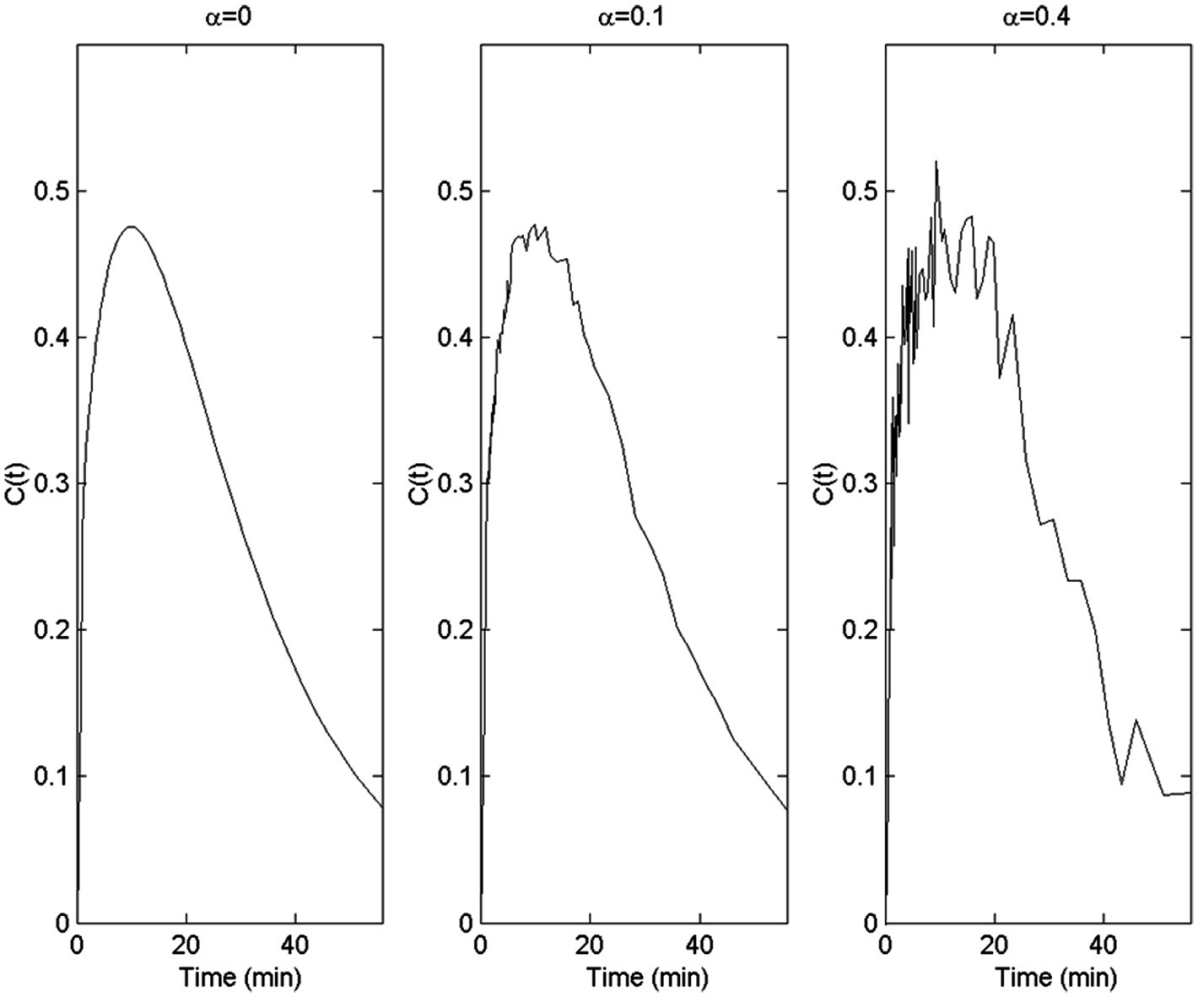
**Time-integrated time-activity curves *C*(*t*) with 3 different noise levels.** The total integration interval is one minute.

In the numerical examples, we chose *K*_1_ = 0.4 min^-1^, *k*_2_ = 0.3 min^-1^, *k*_3_ = 0.2 min^-1^ and *k*_4_ = 0.1 min^-1^. The *k* values used in the computer simulations were tailored from [27] and [32], where a wide range of the *k* values are reported for different patient studies. For example, *K*_1_ can be anywhere from 0.1 to1.2, and *k*_2_ can be from 0.1 to 0.8, and so on. The values of *k*_3_ and *k*_4_ are smaller. Scaled Gaussian noise, *N*(0,1) (mean = 0, standard deviation = 1), was added to the noiseless data *C*(*t*). The noise scaling factor was

(13) $$\alpha \sqrt{\frac{C\left(t\right)2^{-t/T_{1/2}}}{T\left(second\right)}}\text{,}$$

where *T*_1/2_ (=110 min) was the half-life of the isotope, and the proportional constant *α* = 0 corresponds to the noiseless case. This noise model is suggested in [31]. Typical noisy time activity curves *C*(*t*) are shown in Figure 2 for these three α values. In reality, the images are reconstructed at the pre-selected non-uniformly distributed time instances, and the non-uniformly distributed blood and tissue time-activity-curves are obtained.

The solution of (1)-(3) corresponding to $\hat{B}\left(t\right)$ is denoted as *Ĉ(t)*. Let *B*(*t*) and *C*(*t*) denote the corresponding curves after time integration over the 60 sec interval. {$\hat{B}\left(t\right)$, *Ĉ*(*t*)} and {*B*(*t*), *C*(*t*)} satisfy the second order differential equation (4) based on the theory to be discussed in Data integration effects section.

The standard least-squares fitting method was applied to the model (6) using MATLAB® and used the built-in function “nlinfit.” The MATLAB’s built-in function nlinfit allows negative outcomes. MATLAB also has another built-in function lsqnonlin that enforces the non-negativity constraint on the outcomes and is widely used for non-negative parameter fits.

Some numerical examples are shown in Tables 1, 2, and indicate the importance and sensitivity of the iterative parameter estimation algorithm in selection of a proper initial condition. In Table 1, only one noise realization was used for each case, while in Table 2, 250 noise realizations were used for each case. If a bad initial condition is chosen, the results could be totally wrong even under the ideal noiseless situation. In order to overcome this problem, a closed-form Fourier domain estimation is developed in the next section. In Table 1, the true *k* parameters are not the same as the *k* parameters estimated with noiseless (α = 0) data. In the noiseless (α = 0) case, both *B*(*t*) and *C*(*t*) are error free. If the initial conditions are not properly provided, the estimated *k* parameters from the noiseless data are still far away from the true parameters.

**Table 1:** The results of a standard iterative curve-fitting method with a good and a bad initial condition (using 1 noise realization for each case)

**Case**	**Bad initial condition**				**Good initial condition**			
	***K***_**1 **_**(min**^**-1**^**)**	***k***_**2 **_**(min**^**-1**^**)**	***k***_**3 **_**(min**^**-1**^**)**	***k***_**4 **_**(min**^**-1**^**)**	***K***_**1 **_**(min**^**-1**^**)**	***k***_**2 **_**(min**^**-1**^**)**	***k***_**3 **_**(min**^**-1**^**)**	***k***_**4 **_**(min**^**-1**^**)**
Initial	0.70	0.70	0.70	0.70	0.45	0.35	0.25	0.15
α = 0	0.14	−1.62	−5.10	−2.10	0.40	0.30	0.20	0.10
α = 0.1	0.13	−1.65	−5.14	−2.090	0.4	0.28	0.18	0.10
α = 0.4	0.12	−1.64	−5.03	−2.038	0.391	0.26	0.15	0.09
True	0.40	0.30	0.20	0.10	0.40	0.30	0.20	0.10

**Table 2:** The results of a standard iterative curve-fitting method with a good and a bad initial condition (using 250 noise realizations for each case)

**Case**	**Bad initial condition**				**Good initial condition**			
	***K***_**1 **_**(min**^**-1**^**)**	***k***_**2 **_**(min**^**-1**^**)**	***k***_**3 **_**(min**^**-1**^**)**	***k***_**4 **_**(min**^**-1**^**)**	***K***_**1 **_**(min**^**-1**^**)**	***k***_**2 **_**(min**^**-1**^**)**	***k***_**3 **_**(min**^**-1**^**)**	***k***_**4 **_**(min**^**-1**^**)**
Initial	0.70	0.70	0.70	0.70	0.45	0.35	0.25	0.15
α = 0	0.14	−1.62	−5.10	−2.10	0.40	0.30	0.20	0.10
α = 0.1	0.15 ± 0.01	−1.61 ± 0.07	−5.08 ± 0.16	−2.10 ± 0.05	040 ± 0.00	0.30 ± 0.01	0.20 ± 0.02	0.10 ± 0.00
α = 0.4	0.14 ± 0.05	−1.62 ± 0.34	−5.11 ± 0.78	−2.10 ± 0.27	040 ± 0.02	0.31 ± 0.07	0.21 ± 0.06	0.10 ± 0.01
True	0.40	0.30	0.20	0.10	0.40	0.30	0.20	0.10

### The Fourier domain estimation method

By taking the Fourier transform of (4), we have

(14) $$-\omega^{2}\tilde{C}\left(i\omega \right)=x_{1}i\omega \tilde{B}\left(i\omega \right)+x_{2}\tilde{B}\left(i\omega \right)+x_{3}i\omega \tilde{C}\left(i\omega \right)+x_{4}\tilde{C}\left(i\omega \right)\text{,}$$

where $\tilde{C}\left(i\omega \right)$and $\tilde{B}\left(i\omega \right)$are the Fourier transform of *C*(*t*) and *B*(*t*), respectively, and *ω* is the frequency variable. Let *ω* = 0, (14) immediately gives a DC (direct current) gain relationship:

(15) $$k_{2}k_{4}\tilde{C}\left(0\right)=K_{1}\left(k_{3}+k_{4}\right)\tilde{B}\left(0\right)\text{.}$$

In the time domain, (15) is expressed as

(16) $$k_{2}k_{4}\int_{0}^{\infty }C\left(t\right)dt=K_{1}\left(k_{3}+k_{4}\right)\int_{0}^{\infty }B\left(t\right)dt\text{.}$$

If the noise in *B*(*t*) and *C*(*t*) has a zero mean value over time, (16) is not affected by noise.

We now introduce some short-hand notations:

(17) $$\left\{ \begin{array}{l} \tilde{B}=\tilde{B}\left(i\omega \right)\\ \tilde{C}=\tilde{C}\left(i\omega \right)\\ \tilde{D}=i\omega \tilde{C}\left(i\omega \right)\\ \tilde{E}=i\omega \tilde{B}\left(i\omega \right)\\ \tilde{F}=-\omega^{2}\tilde{C}\left(i\omega \right) \end{array}\right.$$

Using these short-hand notations, Eq. (14) becomes

(18) $$x_{1}\tilde{E}+x_{2}\tilde{B}+x_{3}\tilde{D}+x_{4}\tilde{C}=\tilde{F}\text{.}$$

Substituting (15), namely,$x_{4}\tilde{C}\left(0\right)=-x_{2}\tilde{B}\left(0\right)$, into (18) eliminates *x*_4_, and an objective function *H* can be formulated as:

(19) $$H=||x_{1}\tilde{E}+x_{2}\tilde{B}+x_{3}{\tilde{D}}_{r}+x_{4}\tilde{C}-\tilde{F}|{|}^{2}=||x_{1}\tilde{E}+x_{2}\tilde{G}+x_{3}\tilde{D}-\tilde{F}|{|}^{2}\text{,}$$

where

(20) $$\tilde{G}=\tilde{B}-\tilde{C}\tilde{B}\left(0\right)/\tilde{C}\left(0\right)\text{.}$$

In order to find the minimum of the objective function *H*, we set the partial derivatives of *H* to 0 and obtain a set of linear equations as follows.

(21) AX=P

where

(22) A=E˜,E˜ReE˜,G˜ReE˜,D˜ReG˜,E˜G˜,G˜ReG˜,D˜ReD˜,E˜ReD˜,G˜D˜,D˜,

(23) $$X=\left[\begin{array}{c} x_{1}\\ x_{2}\\ x_{3} \end{array}\right]\text{,}$$

(24) P=ReE˜,F˜ReG˜,F˜ReD˜,F˜.

In (22) and (24), “Re” denotes the operation of “taking the real part,” and the inner-product is defined by

(25) $$\langle f,g\rangle =\int_{0}^{\omega_{c}}f\left(\omega \right)g^{*}\left(\omega \right)d\omega \text{.}$$

A closed-form solution can readily be obtained as

(26) $$X=A^{-1}P\text{.}$$

Finally, the two-compartment model kinetic parameters are estimated as

(27) $$\left\{ \begin{array}{l} K_{1}=x_{1}\\ k_{2}=-x_{2}/x_{1}-x_{3}\\ k_{4}=\frac{x_{2}}{k_{2}}\frac{\tilde{B}\left(0\right)}{\tilde{C}\left(0\right)}\\ k_{3}=-k_{2}-k_{4}-x_{3} \end{array}\right.$$

### TAC extrapolation

If a prolonged data acquisition is not practical, the un-measured “tail” activity curve must be estimated by using extrapolation methods. Since the tail decays monotonically and does not contain any peaks, the tail estimation error is well controlled.

The proposed method depends heavily on the DC gain (or the VD value). If the measurement of the TAC *C*(*t*) is terminated before it reaches zero, the VD value still can be estimated [33]. An alternative approach is to estimate the unmeasured *C*(*t*) decay trend as follows.

Here, we suggest one data extrapolation method that uses a summation of a family of exponential functions with different decay constants to approximate the truncated “tail,” under the constraint that the activity curves tend to zero when time approaches infinity. The truncated TAC *C*(*t*) can be approximated by

(28) $$\frac{C\left(t_{\mathrm{end}}\right)}{10}\sum_{n=1}^{10}e^{-\lambda_{n}\left(t-t_{\mathrm{end}}\right)}$$

where *t*_*end*_ is the time when data acquisition stops and λ_*n*_ (*n* = 1, 2, …, 10) are a family of decay constants and are 0.01, 0.02, …, 0.10 in our illustration examples. The motivation to use a family of exponential decays to approximate the unmeasured data is to avoid any bias towards any particular decay rate which is unknown. In computer simulations, C(*t*_*end*_) was the average value of the last 3 measurements.

### Implementation

The computer procedure is discussed in this section. For the two-compartment model, the steps are:

Step 1: Acquire the blood input function *B*(*t*_*n*_) and the compartment time-activity curve *C*(*t*_*n*_), for *n* = 0, 1, 2, …, *N*_*sample*_-1. Here the sampling interval can be non-uniform and *N*_*sample*_ is the total number of samples.

Step 2: Extrapolate the time-activity-curve using the method described in TAC extrapolation section. Linear interpolate the data so that the resultant data sets have a constant sampling interval *T*. The total number of the new data points becomes a much larger number *N*.

Step 3: Evaluate $\tilde{B}\left(0\right)=\sum_{n=0}^{N-1}B\left(nT\right)$ and $\tilde{C}\left(0\right)=\sum_{n=0}^{N-1}C\left(nT\right)$.

Step 4: Use a DFT (Discrete Fourier Transform) or FFT (Fast Fourier Transform) computer routine to calculate the DFT functions $\tilde{B}$ and $\tilde{C}$.

Step 5: Calculate $\tilde{D}\left(ik\Omega \right)=ik\Omega \tilde{C}\left(ik\Omega \right)$, $\tilde{E}\left(ik\Omega \right)=ik\Omega \tilde{B}\left(ik\Omega \right)$, $\tilde{F}\left(ik\Omega \right)=-{\left(k\Omega \right)}^{2}\tilde{C}\left(ik\Omega \right)$, and $\tilde{G}\left(ik\Omega \right)=\tilde{B}\left(ik\Omega \right)-\tilde{C}\left(ik\Omega \right)\frac{\tilde{B}\left(0\right)}{\tilde{C}\left(0\right)}$.

Step 6: Calculate the inner products that appear in (22) and (24) using the inner product definition (25) and a user selected cutoff index *k*_*c*_ , corresponding to the continuous cutoff frequency *ω*_*c*_.

Step 7: Form the matrices ***A*** and ***P*** as shown in (22) and (24).

Step 8: Solve for ***X*** using (26).

Step 9: Finally, use (27) to obtain estimated kinetic parameters *K*_1_ ~ *k*_4_.

The proposed Fourier domain estimation method is in closed-form and does not need an initial condition.

We now present the results of our algorithm applied to the same computer generated data as in the standard iterative curve-fitting method, the outputs of the proposed Fourier domain are shown in Table 3 as the “Stage 1”. In Table 3, only one noise realization is used for each case, while in Table 4, 250 noise realizations are used for each case. Ten low frequency components were used in the Fourier domain calculation, that is, the cutoff index *k*_*c*_ was chosen to be 10. The non-uniformly sampled, one-hour time-activity-curves were extrapolated into two-hour curves, and then linearly interpolated with a constant re-sampling interval of 5 seconds.

**Table 3:** The results of the combined method

**Case**	**Stage 1: Fourier method output**				**Stage 2: Final iterative method output**			
	***K***_**1 **_**(min**^**-1**^**)**	***k***_**2 **_**(min**^**-1**^**)**	***k***_**3 **_**(min**^**-1**^**)**	***k***_**4 **_**(min**^**-1**^**)**	***K***_**1 **_**(min**^**-1**^**)**	***k***_**2 **_**(min**^**-1**^**)**	***k***_**3 **_**(min**^**-1**^**)**	***k***_**4 **_**(min**^**-1**^**)**
α = 0	0.39	0.27	0.17	0.09	0.40	0.30	0.20	0.10
α = 0.1	0.39	0.27	0.11	0.09	0.40	0.28	0.18	0.10
α = 0.4	0.38	0.23	0.14	0.09	0.42	0.35	0.28	0.12
True	0.40	0.30	0.20	0.10	0.40	0.30	0.20	0.10

The output of the proposed closed-form method is used as the initial condition of the iterative method. One noise realization is used.

**Table 4:** The results of the combined method

**Case**	***K***_**1 **_**(min**^**-1**^**)**	***k***_**2 **_**(min**^**-1**^**)**	***k***_**3 **_**(min**^**-1**^**)**	***k***_**4 **_**(min**^**-1**^**)**	***K***_**1**_***(k***_**3**_ ***+ k***_**4**_***) /(k***_**2**_***k***_**4**_***)***	***K***_**1**_***k***_**3**_***/(k***_**2**_***k***_**4**_***)***	***k***_**3**_***/k***_**4**_
α = 0	0.40	0.30	0.20	0.10	4.00	2.67	2.00
α = 0.1	0.40 ± 0.00	0.30 ± 0.01	0.20 ± 0.01	0.10 ± 0.00	4.00 ± 0.02	2.67 ± 0.05	2.01 ± 0.11
α = 0.4	0.40 ± 0.02	0.31 ± 0.07	0.21 ± 0.06	0.10 ± 0.01	4.01 ± 0.08	2.68 ± 0.19	2.07 ± 0.46
True	0.40	0.30	0.20	0.10	4.00	2.67	2.00

The output of the proposed closed-form method is used as the initial condition of the iterative method. The number of noise realization is 250.

Some macro-parameters have special clinical significance. For example, VD indicates volume-of-distribution, (*K*_1_*k*_3_)/(*k*_2_ + *k*_3_) reflects the net uptake, and (*K*_1_*k*_3_)/( *k*_2_*k*_4_) or *k*_3_/ *k*_4_ are related to the binding potential. Therefore, the estimation of some macro-parameters is also reported in the results in Table 4.

It is noticed from Stage 1 of Table 3 that the results are slightly biased even in the noiseless situation. This is because the data are discretely sampled and the continuous Fourier integration must be approximated by a discrete Fourier transform. Another source of error comes from data extrapolation which is required in this Fourier-domain closed-form method. The requirement is that the Fourier integration interval must contain all non-zero activities. Therefore, it is expected that the traditional iterative non-linear fitting method should have less bias, because neither data extrapolation nor interpolation are necessary.

We have mentioned that when a proper initial condition is provided, the standard least-squares fitting usually gives satisfactory results. However, if an inappropriate initial condition is provided, the algorithm may converge to a wrong solution. When the closed-form method was used to obtain a rough estimation and the iterative method was used to fine tune the solution, much better results were obtained as shown in Table 3.

## Discussion

Some issues related to the proposed closed-form formula are discussed below.

### Data integration effects

Averaging of the time-activity curve (TAC) over a time frame does not affect the accuracy of the equations. Because the TAC *C*(*t*) and the input function *B*(*t*) are related by a linear differential equation, for example (4), *C*(*t*) can be expressed as a convolution with *B*(*t*):

(29) $$C\left(t\right)=B\left(t\right)*H\left(t\right)\text{,}$$

where the kernel *H*(*t*) is determined by the system of differential equation. Averaging *C*(*t*) over a time frame is to convolve *C*(*t*) with a box-car window function or unit-step window function *W*(*t*):

(30) $$C_{\mathrm{avg}}\left(t\right)=C\left(t\right)*W\left(t\right)\text{.}$$

Combining the above two convolution expressions yields

(31) $$C_{\mathrm{avg}}\left(t\right)=\left[B\left(t\right)*H\left(t\right)\right]*W\left(t\right)=\left[B\left(t\right)*W\left(t\right)\right]*H\left(t\right)=B_{\mathrm{avg}}\left(t\right)*H\left(t\right)\text{.}$$

which implies that if we integrate the TAC *C*(*t*) and the input function *B*(*t*) over the same time interval, the integrated functions still satisfy the same differential equations.

### Selection of the cut-off frequency

The selection of the cut-off s based on trial-and-error, and is dependent on noise level. Ideally speaking, the cut-off frequency should be chosen as high as possible to include as much information. However, the high frequency components contain more noise than the low frequency components. If the cut-off frequency is selected too high, the estimation will be affected by the noise fluctuation, resulting in different solutions with different noise realizations. If the cut-off frequency is selected too low, even though the estimation is not sensitive to noise, it may contain large bias because the system is not adequately represented. The effect of the cut-off frequency selection is illustrated by the examples in Table 5, where a two-compartment model is considered with noise level α = 0.4.

**Table 5:** **Estimation results for the two-compartment model parameters *K***_**1**_**, *k***_**2**_**, *k***_**3**_**, and *k***_**4**_**with noise level α = 0.4, using different cut-off frequencies and 250 noise realizations**

**Cut-off frequency index *k***_***c***_	***K***_**1 **_**(min**^**-1**^**)**	***k***_**2 **_**(min**^**-1**^**)**	***k***_**3 **_**(min**^**-1**^**)**	***k***_**4**_**(min**^**-1**^**)**	***K***_**1**_***(k***_**3**_ ***+ k***_**4**_***) /(k***_**2**_***k***_**4**_***)***	***K***_**1**_***k***_**3**_***/(k***_**2**_***k***_**4**_***)***	***k***_**3**_***/k***_**4**_
8	0.36 ± 0.05	0.22 ± 0.10	0.11 ± 0.09	0.07 ± 0.03	4.08 ± 0.07	2.24 ± 0.55	1.43 ± 0.77
10	0.37 ± 0.05	0.24 ± 0.11	0.13 ± 0.08	0.08 ± 0.03	4.07 ± 0.07	2.35 ± 0.49	1.58 ± 0.77
15	0.37 ± 0.04	0.23 ± 0.09	0.11 ± 0.08	0.07 ± 0.03	4.07 ± 0.07	2.27 ± 0.51	1.45 ± 0.71
20	0.37 ± 0.03	0.22 ± 0.08	0.10 ± 0.08	0.06 ± 0.03	4.08 ± 0.07	2.24 ± 0.51	1.39 ± 0.65
True	0.40	0.30	0.20	0.10	4.00	2.67	2.00

### Extension to one-compartment or multi-compartment models

In general, if we have *N*, where *N* can be 1, compartments for the tissue model, we have a system of *N* first-order differential equations (which are called state equations) to describe the kinetics [34]. Let **C** be a vector that contains all *N* compartments, *A* be an *N×N* matrix, *D* be an *N×*1 matrix and *E* be a 1*×N* matrix. The system of differential equations can be expressed in a matrix form

(32) $$\frac{dC\left(t\right)}{dt}=AC\left(t\right)+DB\left(t\right)\text{.}$$

The measurable activity is described by the output equation:

(33) $$C\left(t\right)=EC\left(t\right)\text{,}$$

where the *C*(*t*) on the left-hand-side is a scalar. Using the Fourier transform, the system’s transfer function can be obtained as [35]

(34) $$\tilde{H}\left(i\omega \right)=\frac{\tilde{C}\left(i\omega \right)}{\tilde{B}\left(i\omega \right)}=E{\left(i\omega I-A\right)}^{-1}D=\frac{x_{N+1}+x_{N+2}\left(i\omega \right)+\ldots +x_{N+N}{\left(i\omega \right)}^{N-1}}{{\left(i\omega \right)}^{N}-x_{1}{\left(i\omega \right)}^{N-1}-\ldots -x_{N-1}\left(V\right)-x_{N}}\text{,}$$

namely,

(35) $${\left(i\omega \right)}^{N}\tilde{C}\left(i\omega \right)-x_{1}{\left(i\omega \right)}^{N-1}\tilde{C}\left(i\omega \right)-\cdots -x_{N}\tilde{C}\left(i\omega \right)=x_{N+1}\tilde{B}\left(i\omega \right)+x_{N+2}\left(i\omega \right)\tilde{B}\left(i\omega \right)+\cdots +x_{N+N}{\left(i\omega \right)}^{N-1}\tilde{B}\left(i\omega \right)\text{.}$$

Let *ω* = 0, (35) immediately gives a DC gain relation:

(36) $$-x_{N}\tilde{C}\left(0\right)=x_{N+1}\tilde{B}\left(0\right)\text{.}$$

Similar to the case in the Methods and Results section, the coefficients *x*_1_ … *x*_*N*+*N*_ are related to the kinetic parameters. The parameter estimation problem can be achieved by minimizing an objective function similar to (19). By using the DC gain expressed in (36), the total number of unknowns is 2 *N*-1. The unknown coefficients are obtained by solving a least-squares problem to approximate (35). Finally the kinetic parameters are estimated using relations between the coefficients *x*_1_ … *x*_*N*+*N*_ and the kinetic parameters. The proposed method becomes less effective as *N* increases, partially due to the non-linear relationship between the *k* parameters and *x* parameters. For a large *N*, there may not be a closed-form expressions for the *k* parameter for a set of *x’*s.

### Consideration of the input function contamination effect

In the above discussion, we assume that the quantity *C*(*t*) can be measured. In reality, the measured *C*(*t*) may be contaminated by the input function *B*(*t*). The contamination of the tissue by the blood input function is considered in this section. The contamination of the blood by the tissue is more problematic because it may result in a non-identifiable problem. The system’s transfer function or the impulse response function should be revised to reflect this effect. The revised impulse response function becomes

(37) $$h_{\mathrm{revised}}\left(t\right)=\left(1-f_{v}\right)h\left(t\right)+f_{v}\delta \left(t\right),\text{for}\;t\geq 0\text{,}$$

where *δ*(*t*) is the Dirac delta function, and *f*_*v*_ is the fraction of the contamination. Using the two-compartment model as an example, the revised transfer function is

(38) $$\begin{array}{c} {\tilde{H}}_{\mathrm{revised}}(i\omega )=(1-f_{v})\tilde{H}(i\omega )+f_{v}=(1-f_{v})K_{1}\cdot \frac{i\omega +\left(k_{3}+k_{4}\right)}{{\left(i\omega \right)}^{2}+\left(k_{2}+k_{3}+k_{4}\right)\left(i\omega \right)+k_{2}k_{4}}+f_{v}\\ =\frac{-f_{v}\omega^{2}+\left[K_{1}+f_{v}\left(k_{2}+k_{3}+k_{4}-K_{1}\right)\right]i\omega +\left[\left(k_{3}+k_{4}\right)\left(1-f_{v}\right)K_{1}+f_{v}k_{2}k_{4}\right]}{-\omega^{2}+\left(k_{2}+k_{3}+k_{4}\right)i\omega +k_{2}k_{4}}\text{.} \end{array}$$

If we follow the same steps as outlined in Methods and Results section and treat *f*_*v*_ as another unknown parameter, closed-form formulas for the kinetic parameters as well as *f*_*v*_ can be obtained.

### Relation to other linear methods

Generally speaking, a frequency-domain method is equivalent to a time-domain method. Using a cut-off in the frequency-domain is equivalent to performing smoothing in the time-domain. However, the discretization can introduce some complications. The time domain approximation of the derivative *B*’(*t*) by finite difference $\left[B\left(t+\Delta t\right)-B\left(t\right)\right]/\Delta T$is not quite equivalent to $ik\Omega \tilde{B}\left(ik\Omega \right)$ in the Fourier domain. All linear methods such as LLS and GLLS have their different ways to converting differential equations into algebraic equations. The goal is to remove the (continuous) derivative operator, which cannot be implemented directly with sampled data. In the LLS and GLLS method, the derivative operator is removed by integrating both sides of the differential equation. In the proposed Fourier domain method, the derivative operator is indirectly implemented in the Fourier domain as a multiplication of *ik*Ω. These two approaches may not be equivalent.

### Special treatment of the DC component

We give the DC component special attention because of noise considerations. When the noise is zero-mean, the DC-component is almost un-affected; however, all other frequency components are affected. In a system of equations, we trust the DC relationship the most and enforce it to be a constraint. We discard the noise-heavily-affected components (i.e., the high frequency components). We then weigh the remaining frequency components evenly, as an un-weighted least-square objective function. We control the noise by dividing the frequency components in three categories: Trustworthy (i.e., the DC), somewhat trustworthy (i.e., the low-frequencies), and not-trustworthy (i.e., the high frequencies). In cases where the initial estimate of VD is more problematic than in our current simulations, it is possible to remove the VD constraint and use one more unknown variable in the formulation of the closed-form solution.

We have made another version of the analytical method, in which we do not use the DC gain as a constraint. The DC term is treated almost the same as other low-frequency components but with 1000 times heavier weights. In our simulations, the resultant algorithm was not as stable as the proposed algorithm, in which the DC gain is used as a constraint.

## Conclusions

Time-domain curve-fitting is the current state-of-the-art in nuclear medicine kinetic estimation. Due to the non-linear exponential functions, this curve-fitting is sensitive to initial conditions (i.e., the initial solutions) for multi-compartment model parameter estimation problems. The initial condition may make the algorithm converge to a wrong solution. In this paper, a Fourier-domain kinetic estimation method is proposed. The proposed Fourier-domain curve-fitting method does not require an initial condition, it minimizes a quadratic objective function and a closed-form solution can be obtained. The noise is easier to control, simply by discarding the high frequency components, and emphasizing the DC component. The proposed Fourier-domain estimation method has closed-form formulas.

The unique strategy in this paper is to formulate the objective function in the Fourier domain. This strategy has two advantages: The model is linear in terms of transformed variables (18) and it is easier to control noise by discarding high frequency components. The Frequency domain objective function is a quadratic function. To minimize it, the gradients are set to zero. This results in a system of linear equations with a small number of unknowns.

If the time signals are truncated during data acquisition when the signal values have not reached a very small value, the unmeasured “tails” must be extrapolated and appended to the measured signals before the Fourier transform is taken. However, data extrapolation and interpolation may introduce errors. For irreversible tracers, the activity curve does not decay to zero at all while the input function decays to zero. Our proposed method does not apply and should be modified by using the derivatives of the time-activity-curves to replace the time-activity-curves.

To compare the standard non-linear iterative curve-fitting technique and the closed-form linear estimation methods, the iterative curve-fitting technique is more robust in a noisy environment but is strongly dependent on the initial conditions. Large bias on the estimated kinetic parameters (i.e., the *k* values) is common if the initial condition is not close enough to the true solution.

Many linear estimation methods are available. The linear coefficients are formed by the measured data and are thus influenced by noise. The linear estimation of the parameters is, in general, biased and noisy, even though no initial condition is needed.

For the two compartment model, the proposed method is sensitive to noise. Thus the result is not very accurate. The advantage of the proposed method is that the user does not need to specify the initial condition. On the other hand, the traditional curve-fitting method strongly depends on the initial condition. Therefore, the proposed method can be used to provide the initial condition that is close to the true solution, and then the traditional curve-fitting method is used to fine-tune the parameter estimation.

## Competing interests

The authors declare that they have no competing interests.

## Authors’ contributions

All authors read and approved the final manuscript.

## References

[B1] Cherry SR Sorenson JA Phelps ME Physics in Nuclear Medicine 2003 3 Philadelphia: Saunders

[B2] Phelps ME PET: Molecular Imaging and Its Biological Applications 2004 New York: Springer Science

[B3] Watabe H Ikoma Y Kimura Y Naganawa M Shidahara M PET kinetic analysis—compartmental model Ann Nucl Med 2006 20 583 588 10.1007/BF02984655 17294668

[B4] Gullberg GT Reutter BW Sitek A Maltz J Budinger TF Dynamic single photon emission computed tomography — basic principles and cardiac applications Phys Med Biol 2010 55 R111 R191 10.1088/0031-9155/55/20/R01 20858925 PMC3306016

[B5] Press WH Flannery BP Teukolsky SA Vetterling WT Numerical Recipes in C 1988 Cambridge: Cambridge University Press

[B6] Byrtek M O'Sullivan F Muzi M Spence AM An adaptation of ridge regression for improved estimation of kinetic model parameters from PET studies IEEE 2003 Nuclear Science Symposium Conference Record 2003 3120 3124

[B7] Byrtek M O'Sullivan F Muzi M Spence AM An adaptation of ridge regression for improved estimation of kinetic model parameters from PET studies IEEE Trans Nucl Sci 2005 52 63 68

[B8] O'Sullivan F Saha A Use of ridge regression for improved estimation of kinetic constants from PET data IEEE Trans Med Imaging 1999 18 115 125 10.1109/42.759111 10232668

[B9] Yun Z Sung-Cheng H Bergsneider M Linear ridge regression with spatial constraint for generation of parametric images in dynamic positron emission tomography studies IEEE Trans Nucl Sci 2001 48 125 130 10.1109/23.910842

[B10] Zhou Y Huang SC Bergsneider M Wong DF Improved parametric image generation using spatial-temporal analysis of dynamic PET studies Neuroimage 2002 15 697 707 10.1006/nimg.2001.1021 11848713

[B11] Zhou Y Endres CJ Brasic JR Huang SC Wong DF Linear regression with spatial constraint to generate parametric images of ligand-receptor dynamic PET studies with a simplified reference tissue model Neuroimage 2003 18 975 989 10.1016/S1053-8119(03)00017-X 12725772

[B12] Wong KP Meikle SR Feng D Fulham MJ Estimation of input function and kinetic parameters using simulated annealing: application in a flow model IEEE Trans Nucl Sci 2002 49 707 713 10.1109/TNS.2002.1039552

[B13] Yaqub M Boellaard R Kropholler MA Lammertsma AA Optimization algorithms and weighting factors for analysis of dynamic PET studies Phys Med Biol 2006 51 4217 4232 10.1088/0031-9155/51/17/007 16912378

[B14] Blomqvist G On the construction of functional maps in positron emission tomography J Cereb Blood Flow Metab 1984 4 629 632 10.1038/jcbfm.1984.89 6334095

[B15] Carson RE Huang SC Green ME Weighted integration method for local cerebral blood flow measurements with positron emission tomography J Cereb Blood Flow Metab 1986 6 245 258 10.1038/jcbfm.1986.38 3485644

[B16] Yokoi T Kanno I Iida H Miura S Uemura K A new approach of weighted integration technique based on accumulated images using dynamic PET and H2(15)O J Cereb Blood Flow Metab 1991 11 492 501 10.1038/jcbfm.1991.93 2016358

[B17] Chen K Lawson M Reiman E Cooper A Feng D Huang SC Bandy D Ho D Yun LS Palant A Generalized linear least squares method for fast generation of myocardial blood flow parametric images with N-13 ammonia PET IEEE Trans Med Imaging 1998 17 236 243 10.1109/42.700735 9688155

[B18] Feng D Ho D Lau KK Siu WC GLLS for optimally sampled continuous dynamic system modeling: theory and algorithm Comput Methods Programs Biomed 1999 59 31 43 10.1016/S0169-2607(98)00099-6 10215175

[B19] Ho D Feng D Rapid algorithms for the construction of cerebral blood flow and oxygen utilization images with oxygen-15 and dynamic positron emission tomography Comput Methods Programs Biomed 1999 58 99 117 10.1016/S0169-2607(98)00069-8 10092026

[B20] Wen L Eberl S Fulham MJ Feng DD Bai J Constructing reliable parametric images using enhanced GLLS for dynamic SPECT IEEE Trans Biomed Eng 2009 56 1117 1126 19068420 10.1109/TBME.2008.2009998

[B21] Boellaard R Knaapen P Rijbroek A Luurtsema GJ Lammertsma AA Evaluation of basis function and linear least squares methods for generating parametric blood flow images using 15O-water and Positron Emission Tomography Mol Imaging Biol 2005 7 273 285 10.1007/s11307-005-0007-2 16080023

[B22] Gunn RN Gunn SR Turkheimer FE Aston JA Cunningham VJ Positron emission tomography compartmental models: a basis pursuit strategy for kinetic modeling J Cereb Blood Flow Metab 2002 22 1425 1439 12468888 10.1097/01.wcb.0000045042.03034.42

[B23] Hong Y T and Fryer T D: Kinetic modelling using basis functions derived from two-tissue compartmental models with a plasma input function: general principle and application to [18 F]fluorodeoxyglucose positron emission tomography Neuroimage 2010 51 164 172 10.1016/j.neuroimage.2010.02.013 20156574

[B24] Reader AJ Sureau FC Comtat C Trebossen R Buvat I Joint estimation of dynamic PET images and temporal basis functions using fully 4D ML-EM Phys Med Biol 2006 51 5455 5474 10.1088/0031-9155/51/21/005 17047263

[B25] Verhaeghe J Van de Ville D Khalidov I D'Asseler Y Lemahieu I Unser M Dynamic PET reconstruction using wavelet regularization with adapted basis functions IEEE Trans Med Imaging 2008 27 943 959 18599400 10.1109/TMI.2008.923698

[B26] Watabe H Jino H Kawachi N Teramoto N Hayashi T Ohta Y Iida H Parametric imaging of myocardial blood flow with 15O-water and PET using the basis function method J Nucl Med 2005 46 1219 1224 16000292

[B27] Feng D Ho D Chen K Wu L-C Wang J-K Liu R-S Yeh S-H An evaluation of the algorithms for determining local cerebral metabolic rates of glucose using positron emission tomography dynamic data IEEE Trans Med Imaging 1995 14 697 710 10.1109/42.476111 18215874

[B28] Dai X Chen Z Tian J Performance evaluation of kinetic parameter estimation methods in dynamic FDG-PET studies Nucl Med Commun 2011 32 4 16 10.1097/MNM.0b013e32833f6c05 21166088

[B29] Zeng GL Kadrmas DJ Gullberg GT Fourier domain closed-form formulas for estimation of kinetic parameters in multi-compartment models 2011 IEEE Nuclear Science Symposium and Medical Imaging Conference (NSS/MIC 2011) 3209 3216

[B30] Zeng GL Gullberg GT Kadrmas DJ Closed-form kinetic parameter estimation solution to the truncated data problem Phys Med Biol 2010 55 7453 7468 10.1088/0031-9155/55/24/005 21098917 PMC3023984

[B31] Feng D Huang SC Wang X Models for computer simulation studies of input functions for tracer kinetic modeling with positron emission tomography Int J Biomed Comput 1993 32 95 110 10.1016/0020-7101(93)90049-C 8449593

[B32] Oriuchi N Tomiyoshi K Ahmed K Sarwar M Tokunaga M Suzuki H Watanabe N Hirano T Shibasaki T Tamura M Endo K Independent Thallium-201 accumulation and Fluorine-18-Fluorodeoxyglucose metabolism in glioma J Nucl Med 1996 37 457 462 8772644

[B33] Ichise M Toyama H Innis RB Carson RE Strategies to improve neureceptor parameter estimation by linear regression analysis J Cereb Blood Flow Metab 2002 22 1271 1281 12368666 10.1097/01.WCB.0000038000.34930.4E

[B34] Gunn RN Gunn SR Cunningham VJ Positron emission tomography compartmental models J Cereb Blood Flow Metab 2001 21 635 652 11488533 10.1097/00004647-200106000-00002

[B35] Franklin GF Powell JD Emami-Naeini A Feedback Control of Dynamic Systems 2002 4 Upper Saddle River, New Jersey: Prentice-Hall Inc

